# Using Business Intelligence to Analyze and Share Health System Infrastructure Data in a Rural Health Authority

**DOI:** 10.2196/medinform.3590

**Published:** 2014-08-06

**Authors:** Waqar Haque, Bonnie Urquhart, Emery Berg, Ramandeep Dhanoa

**Affiliations:** ^1^University of Northern British ColumbiaDepartment of Computer Science and School of BusinessPrince George, BCCanada; ^2^Northern HealthPlanning and Performance ImprovementPrince George, BCCanada; ^3^University of Northern British ColumbiaDepartment of Computer SciencePrince George, BCCanada

**Keywords:** business intelligence, health care systems, availability of health services, data visualization

## Abstract

**Background:**

Health care organizations gather large volumes of data, which has been traditionally stored in legacy formats making it difficult to analyze or use effectively. Though recent government-funded initiatives have improved the situation, the quality of most existing data is poor, suffers from inconsistencies, and lacks integrity. Generating reports from such data is generally not considered feasible due to extensive labor, lack of reliability, and time constraints. Advanced data analytics is one way of extracting useful information from such data.

**Objective:**

The intent of this study was to propose how Business Intelligence (BI) techniques can be applied to health system infrastructure data in order to make this information more accessible and comprehensible for a broader group of people.

**Methods:**

An integration process was developed to cleanse and integrate data from disparate sources into a data warehouse. An Online Analytical Processing (OLAP) cube was then built to allow slicing along multiple dimensions determined by various key performance indicators (KPIs), representing population and patient profiles, case mix groups, and healthy community indicators. The use of mapping tools, customized shape files, and embedded objects further augment the navigation. Finally, Web forms provide a mechanism for remote uploading of data and transparent processing of the cube. For privileged information, access controls were implemented.

**Results:**

Data visualization has eliminated tedious analysis through legacy reports and provided a mechanism for optimally aligning resources with needs. Stakeholders are able to visualize KPIs on a main dashboard, slice-and-dice data, generate ad hoc reports, and quickly find the desired information. In addition, comparison, availability, and service level reports can also be generated on demand. All reports can be drilled down for navigation at a finer granularity.

**Conclusions:**

We have demonstrated how BI techniques and tools can be used in the health care environment to make informed decisions with reference to resource allocation and enhancement of the quality of patient care. The data can be uploaded immediately upon collection, thus keeping reports current. The modular design can be expanded to add new datasets such as for smoking rates, teen pregnancies, human immunodeficiency virus (HIV) rates, immunization coverage, and vital statistical summaries.

##  Introduction

Health care extends beyond medicine in many ways. One of these is the ability to access health care services, particularly when one is located far from the core infrastructure. Access to relevant information in an intuitive form not only benefits the patient, but also assists the administration in identifying areas where resource allocation may have the highest impact. This ultimately leads to healthier communities and optimal use of health care funding. Fortunately, large volumes of information have been gathered over the years and this serves as a base for achieving the envisioned goals. Despite many recent government-funded initiatives, much of this information sits in legacy formats and the sheer volume of data makes it incomprehensible for any use other than the specific purpose for which each dataset was gathered. In addition, the data is of poor quality, suffers from inconsistencies, and lacks integrity. Despite having the data, health care providers and supporting staff are faced with the challenge of determining the type and location of resources accessible to them and their patients. In order to locate this information, an extensive search through numerous Microsoft Excel workbooks, databases, and statistical websites is quite common. Even then, it can be extremely tedious to find the needed information from these sources because they generally differ in purpose and tend to be inconsistent with each other.

Traditionally, Business Intelligence (BI) has been used to analyze business information such as marketing and/or financial reporting data. In this paper, we propose how BI techniques can be applied to health care infrastructure data in order to make this information more accessible and comprehensible for a broader group of people. Our envisioned goal has been achieved by first consolidating the sources into a singular entity, second providing interactive access and control of the underlying data, and finally visually representing the data through reports. By applying these techniques, the resulting information can be accessed through dashboards, which provide a quick overview of the key performance indicators (KPIs) and allow navigation to underlying reports of finer granularity. Thus, instead of sifting through massive spreadsheets for the desired information, one can now access a centralized system that renders reports in a matter of seconds. The system also extends to other tools such as Web forms for updating data by designated staff without the need for going through complex IT protocols. The underlying data represents geography and services for the entire region covered by Northern Health (NH), that is, a population of approximately 300,000, land mass of roughly 600,000 km^2^, and the breadth of services from health prevention and promotion through to acute care services. Northern Health is located in British Columbia, Canada, and is one of the seven health authorities in the province responsible for delivery of publicly funded health services. Five of the health authorities are based on geography, one is responsible for province-wide tertiary services, and one is responsible for First Nations health services. The Canadian health care system is publicly funded for the most part, with funding from both the federal government and the provincial or territorial governments.

## Methods

### Data Integration, Analysis, and Reporting

**Figure 1:**
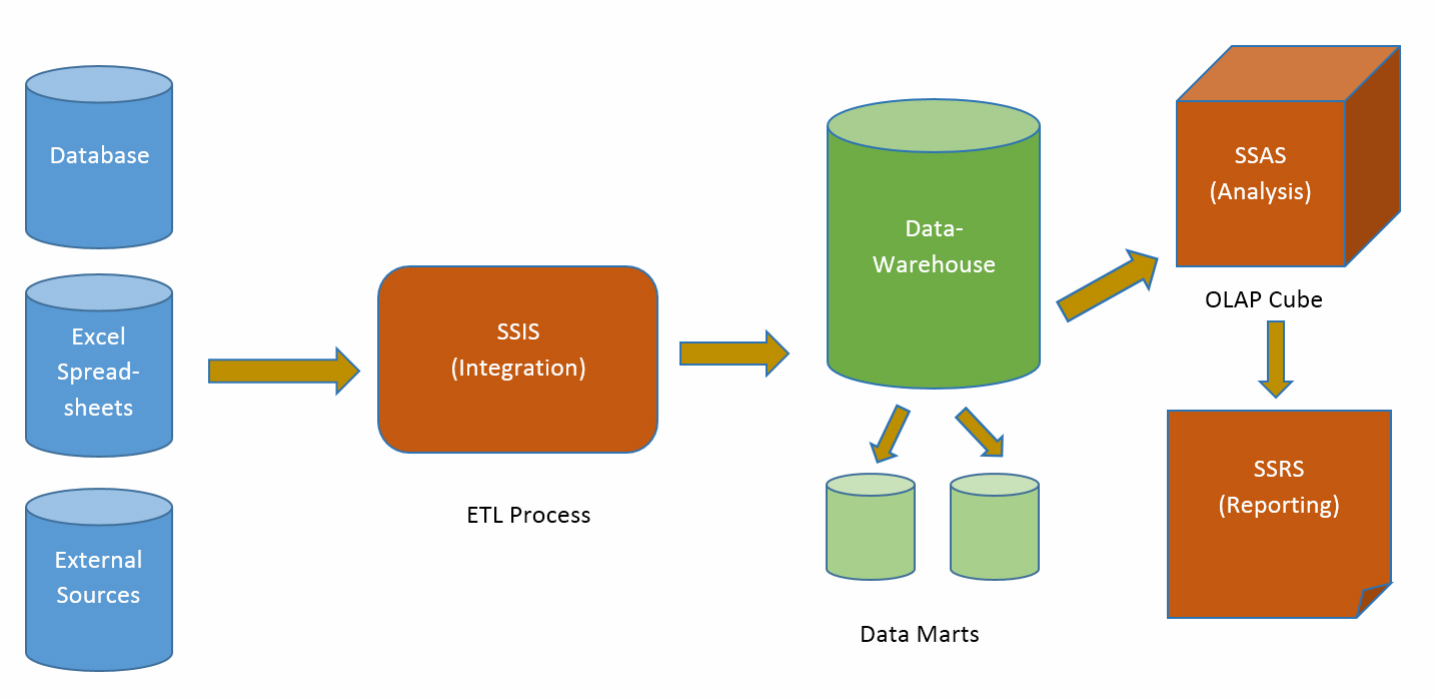
Business intelligence modeling overview.

Business intelligence tools and techniques are an effective way to integrate and analyze large data repositories. However, the integration process becomes challenging when the data is not collected with analytics in mind. In our solution, we used Microsoft SQL Server’s BI tool stack [1] and Web development framework, ASP.NET [2], to make the data more accessible and reduce the time that data analysts spend searching through large collections of sources. We also merged the disparate data sources to eliminate data conflicts and create a singular source for reporting. The resulting data warehouse became the central source for all analysis and reporting (Figure 1). An extract-transform-load (ETL) [3] process was used to populate the data warehouse. During the extract phase, connections were created to various data sources and the required information was pulled into temporary storage. In the transform phase, the format of stored data was made consistent with metadata prior to loading into the data warehouse. The SQL Server Integration Services (SSIS) component in Microsoft’s BI tool stack was used to accomplish this integration using an ETL process. SSIS provides the ability to fetch data from disparate sources and apply different transformations on the data, for example, convert from one data type to another, alter data in the sorted order, etc. An Online Analytical Processing (OLAP) cube was then created using the Analysis Services. This cube is an n-dimensional structure, which can be used to reveal more complex details at various levels of granularity through predesigned and ad hoc queries. The cube consists of several dimensions and fact tables [3]. Using the cube structure, reports are created and rendered through Microsoft’s reporting services [1]. SQL Server Reporting Services provides a rich set of data visualization features such as charts, tables, matrices, gauges, maps, and tooltips. A dashboard gives a high-level overview of the KPIs and acts as a central navigation hub to other reports. Mapping allows the user to see the information based on regions and provides visual representation of distances between locations. Web forms are used to allow users to remotely update information with automatic consistency checks. Normally, updates to a database require knowledge of the underlying structure and the associated query language. By providing a Web form, these queries are created automatically and the database structure is represented visually for easy understanding.

### Related Work

Historically, the health care field has been slow to adopt new computer technologies; this has been largely due to hardware limitations, insufficient computer literacy, mechanical user interfaces, and privacy concerns. The first two causes have been mostly overcome due to the penetration of computers in daily lives and the technological advancements in computer hardware, but many applications are still mechanical in nature and are not intuitive to the user [4]. The Minnesota Health Association developed a pilot program to combine clinical information with administrative data, which faced many challenges such as the expertise of those involved and communication issues resulting from distributed data sources [5]. BI tools and techniques have been used to provide insight into ambulatory care sensitive conditions within Northern Health by analyzing data and identifying areas that need attention [6]. These techniques have also been used successfully to improve the management of large quantities of medical information [7]. Historical information and comparisons with the United States’ primary care system has shown that providing improved access to primary care reduces the cost of health care and enhances the care provided to patients [8]. A comparison survey observed that with the increased access to health care in Canada, the general health of the public was superior to that in the United States [9]. Another study showed that though Canada has a relatively lower cost of health care, the wait times negatively affect the perceived availability of care [10]. Additionally, disparities in health care and its access have been shown to be negatively related to lower income, education, and race both due to perception and access [11]. There has been little or no significant evidence of work that incorporates the concept of BI in analysis of data related to asset mapping or services availability.

### Data Challenges and Cube Design

The underlying data was collected over several years for varying purposes including generation of community health reports. The complexity of the underlying data posed several challenges in the integration phase. The first and foremost challenge was the sheer number of workbooks (a workbook can have many Microsoft Excel spreadsheet files), which have been the primary source of information for the data analysts for several years. An initial screening eliminated irrelevant data, but even after this exercise a very large number of workbooks remained. Most of these contained several sheets that were created for a variety of (sometimes unrelated) purposes, which meant the data did not always match in content or level of granularity. Even the repeated numbers sometimes differed across the workbooks. The data was available at various levels of hierarchy making aggregations unpredictable. Similarly, different naming schemes were used for locations without specifying any clear relationship(s) among them. To deal with this, fuzzy lookups [3] were used by specifying a threshold to match names that are similar enough but not identical. For locations that failed to match, a manual mapping table was created and the names were corrected at the database level through SQL queries.

**Figure 2:**
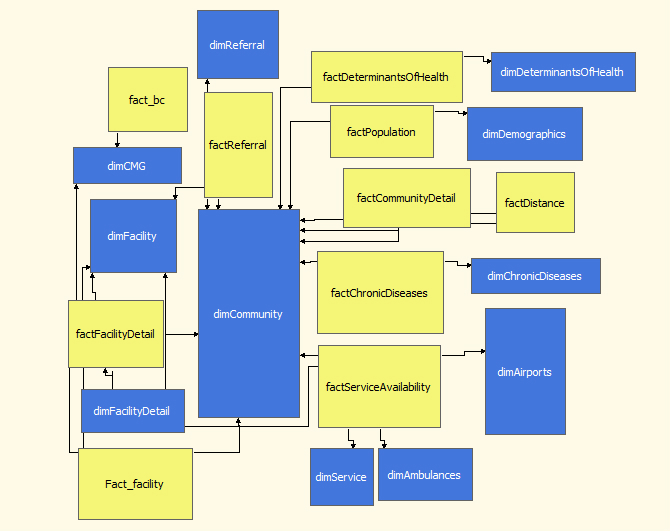
The Online Analytical Processing (OLAP) cube.

A relational model was developed to create a singular source of information. This database consisted of 24 relations, which were populated by three dump sheets via Web forms (described later). An integration package was built to cleanse, combine, and group the data based on its purpose and granularity. When there were conflicts due to repeated information, the selection was based on conformity with other sources and the age of data. In rare cases, informed calculations were performed to correctly reflect missing values. The next step was to create an analysis cube using this database. Normally such cubes use star schema with a single fact table and multiple dimensions [3]. While this structure gives superior performance due to a reduced need for joins, it requires all information to exist at the same level of granularity. This was impractical in our case because of the need for added rows and empty cells if the data were to be restructured. Thus, in our somewhat unusual design, 10 fact tables and 11 dimensions were used (Figure 2), primarily for performance and granularity reasons.

Another challenge was to allow seamless update of data by analysts and staff unfamiliar with the underlying schemas and not trained to write sophisticated queries. Besides having a capability for bulk loading of large volumes of data, there was also a need for the ability to update individual rows without affecting the integrity of the database. To provide this functionality, a Web form was created in ASP.NET [2]. An intuitive combination of tabs, groupings, and dropdown lists allow data entry into individual cells of the selected table. The entered values were checked against metadata before updates were committed. Another mechanism to prevent inconsistencies was the use of dropdown lists when names were referenced. For bulk loading, two dump sheets reflecting the database structure were created. These sheets allow data to be compiled or manually entered and uploaded to the Web form. An integration process then triggers to transparently upload the data and reprocess the cube. The integration is fast, stable, and reliable due to instant validation and simplified logic. The data was also validated by sending reports to administrators of relevant health service delivery areas and by checking against existing manually generated reports.

## Results

### Reporting

For interactive access to information and data visualization, Microsoft SQL Reporting Services [1] were used to generate dynamic reports. In addition to conventional charts and graphs, access to advanced features like mapping, navigational controls, and parameterization of reports is also provided. The information contained in these reports can be updated through the Web form, which automatically reprocesses the cube and immediately reflects the changes.

### Main Dashboard

**Figure 3:**
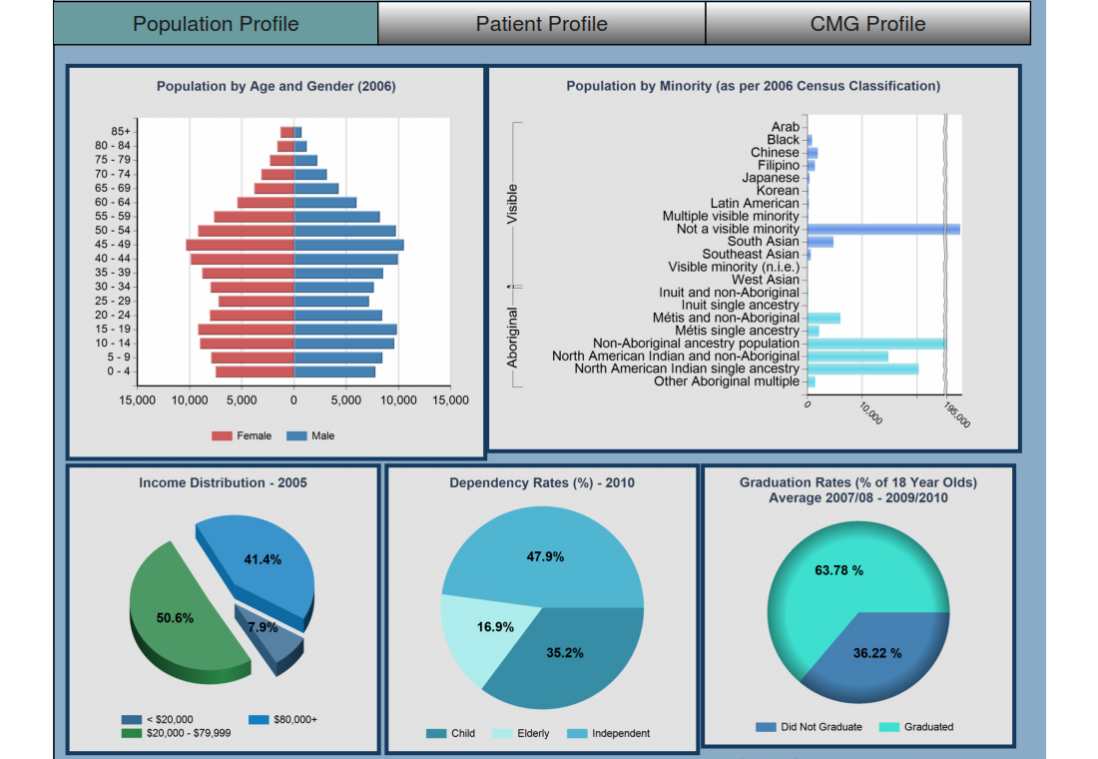
Main dashboard: Population profile.

**Figure 4:**
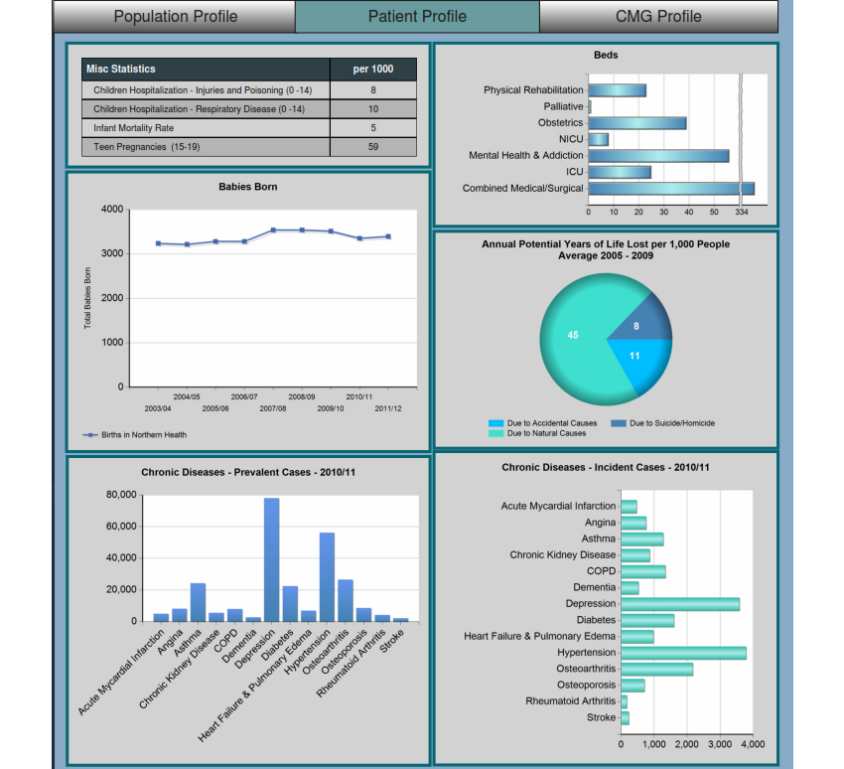
Main dashboard: Patient profile.

**Figure 5:**
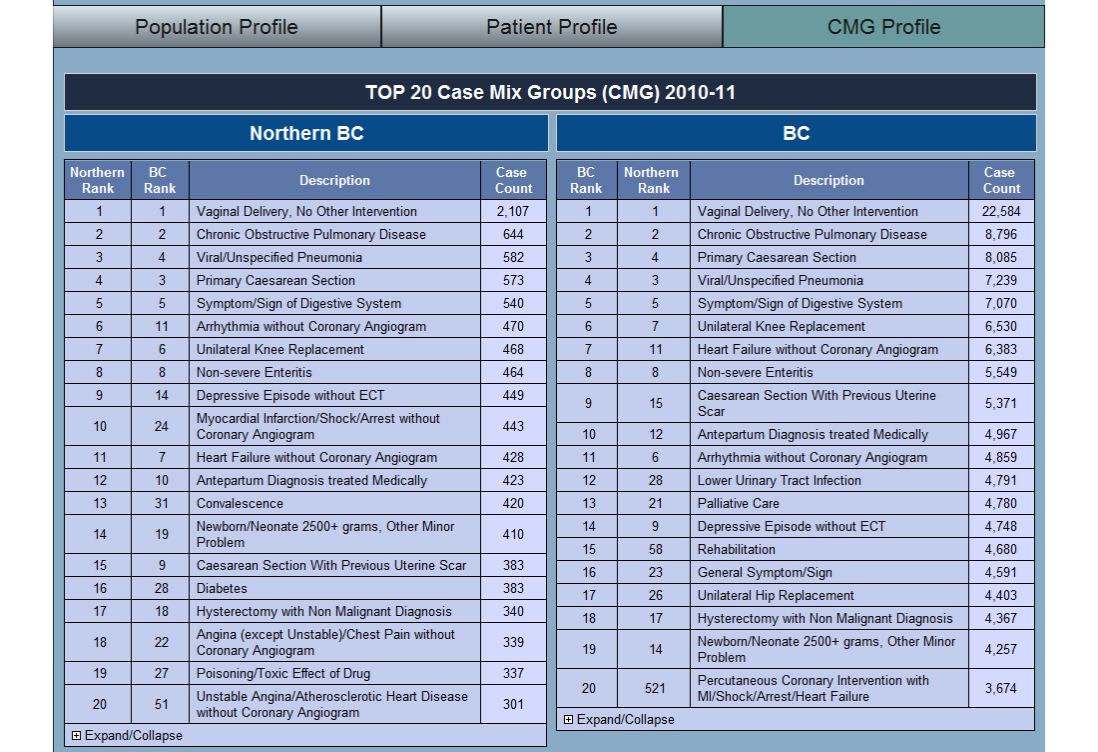
Main dashboard: Case Mix Groups (CMG) profile.

The main dashboard provides an overview of the KPIs and includes navigation controls including a toggle control to switch between demographic information, patient profiles, and case mix groups (CMG). The demographic information displays information relevant to the population status including factors such as wealth, education level, origin [12], and dependency rates (Figure 3). These metrics assist in identifying potential areas of concern and any needed level of support and services. The patient profile gives an overview of the health-related metrics within the selected region by showing information such as births, commonality of chronic conditions, and vaccine preventable diseases (Figure 4). The CMG profile ranks the top 20 reasons for hospitalization in the selected geographic region as compared with the entire province (Figure 5).

This information is available at all levels of hierarchy with the granularity becoming finer from Northern Health Authority (NHA) to Health Service Delivery Areas (HSDA) to Local Health Authorities (LHA) to Communities. This hierarchy can be selected from the maps, which in turn generates the parameters for necessary filtration of information. Tabbed controls allow switching to other reports while maintaining the current level of hierarchy. These tabs allow access to subgroups such as availability of services, comparisons of selected regions, service levels, and direct access to community profiles. To improve navigational performance, the header is embedded with parameters to track the current tab select, type of report (drill-down), and the current level. This allows transparent passing of parameters to determine which report or level to load next based on the direction of navigation.

### Critical Care Dashboard

**Figure 6:**
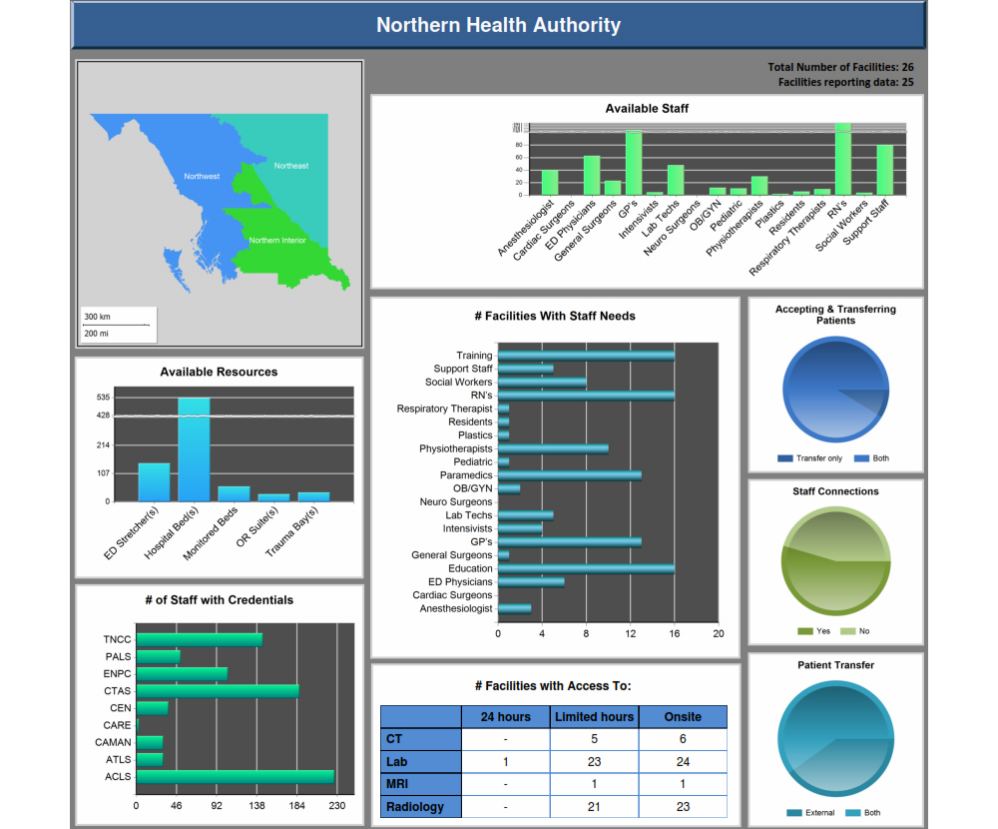
Critical Care dashboard.

**Figure 7:**
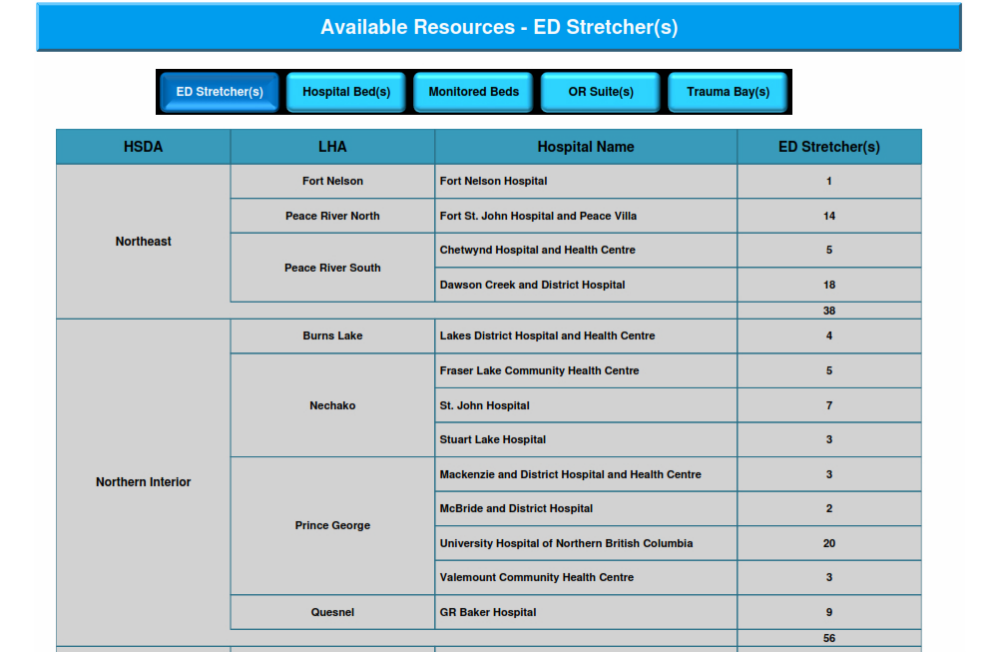
Available resources - drilldown report.

The Critical Care dashboard is an extension of the main dashboard; however, as the combined project progressed it became necessary for the two to split. The reasons for the split included level of granularity desired, validation of data by different groups, and the inward facing (to institution) nature of the Critical Care dashboard as opposed to the outward facing (to public) for the Services Availability dashboard. This dashboard shows the availability of resources for the hospitals and health centers. The ideas and approaches used in the main dashboard have been carried over to the Critical Care dashboard. It opens up to an overview of the NHA area, having drill-down capabilities to HSDA and then to LHA level through a map (Figure 6). Main metrics of the dashboard are Available resources and staff, Number of hospitals with staffing needs, Accepting and transferring patients, Staff credentials and connections, Bed-line transfer use, Airway support, Ventilator capacity, Ventilating patients, Respirators, Ambulances, First Nation Communities, etc. Since all the facilities do not input data, the number of facilities reporting data is described at the top right side of the page. All metrics on this page have drill-down capabilities for easier analysis (Figure 7).

### Mapping Functionality

**Figure 8:**
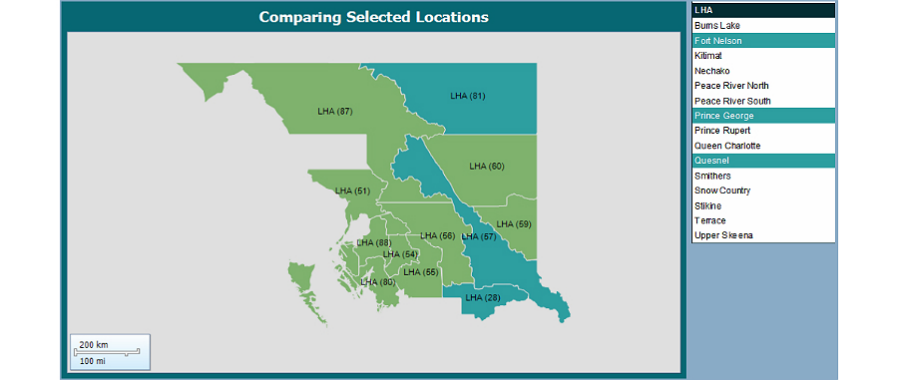
Comparison map.

The mapping features allow location-based visualization and navigation. A challenge, however, was the lack of availability of full range of maps. While default maps are provided by the tools, no maps of British Columbia (BC) were included; this resulted in the need for a shape file to store geographic information such as the shape of regions, locations of communities, or other geographic features. The shape files of BC were obtained from [13] and modified to better fit our needs. These modifications were done through an open source geographic information system (GIS) application, QuantumGIS [14]. Using this application, the shape file of BC was restricted to the area covered by NH; another shape file was created for storing the locations of communities within the region. To address stability issues created by the large size of the single shape file, steps were taken to limit the information contained therein, primarily by removing information that was available elsewhere. Maps were also used for controls in the comparison report and to visualize availability of services in the community or proximity. An example of these controls can be seen in Figure 8 where the map has been used to select LHAs for comparisons.

### Comparison

**Figure 9:**
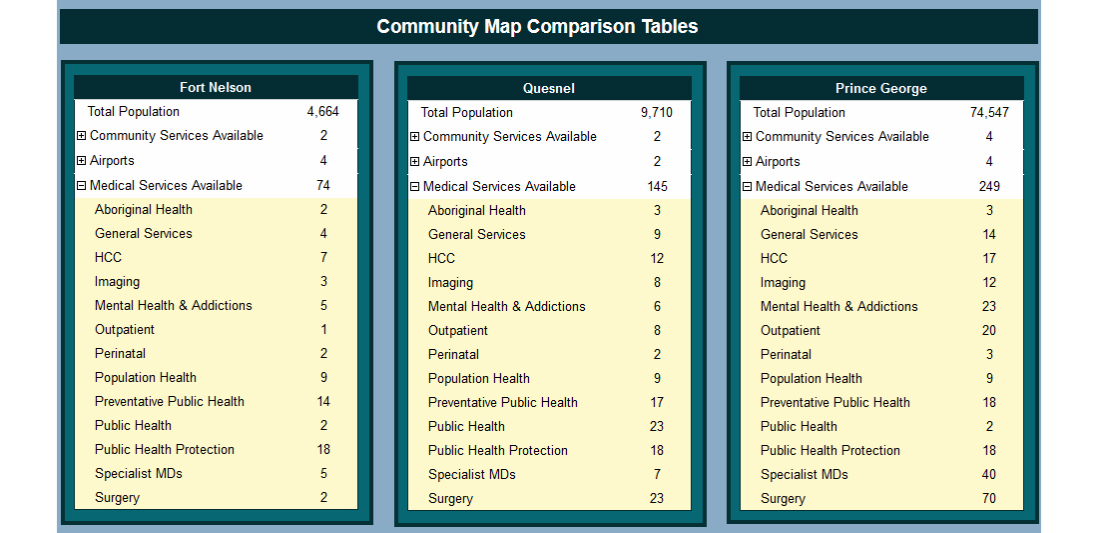
Community comparison tables.

Comparisons between differing regions provide further insight into the state of health care within a region and potential causes for disparities [9]. The regions of interest can be selected by simply clicking on the map. The comparison can be performed at all levels and allows for comparing up to three regions at a time (Figure 8). When a new region is selected for comparison, the three most recent selections are maintained. Metrics such as population, facilities, community services (airports, ambulances, etc), and medical services are displayed for each selected region (Figure 9).

### Service Availability

**Figure 10:**
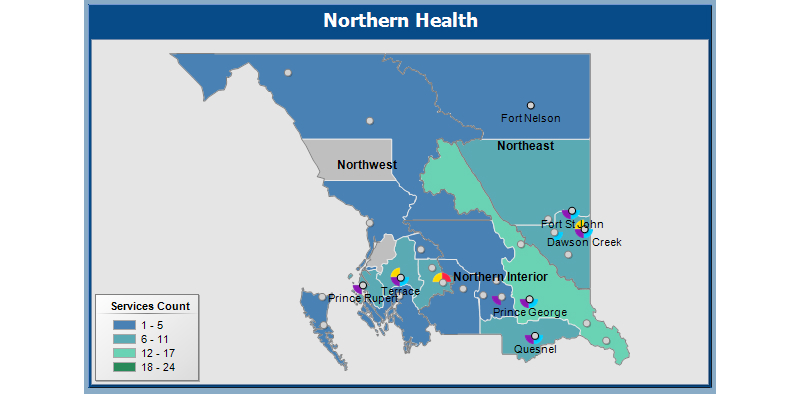
Available map.

**Figure 11:**
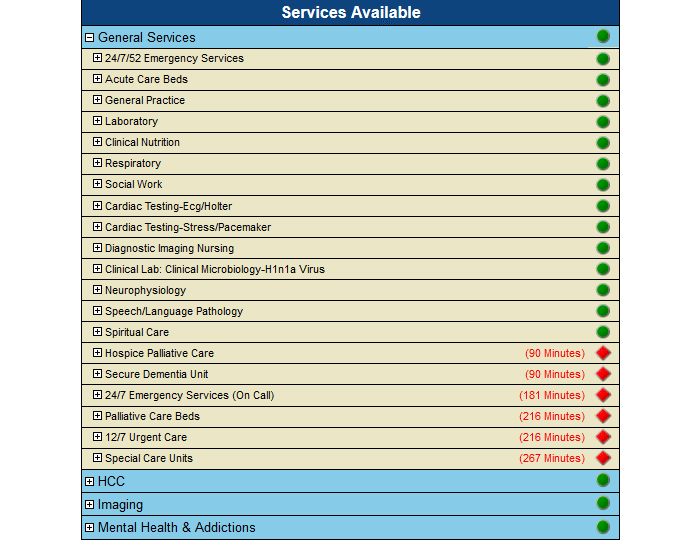
Community profile: Service availability.

Examining the demographic information, patient details, and availability of services has been shown to be effective in identifying possible needs of a region and improving the care of patients [8,10,11,15]. This information has been provided using color-coded markers on the map. For readability purposes, we show up to four services in circles split into quarters (Figure 10). The services are selected from a categorized list; additional information about the locations is displayed through tooltip display when hovering over the circles. Each time a service is selected, the map is updated to show the communities that have facilities offering the selected services. All services offered in NH, whether or not those are available locally, can also be seen in the availability report at the community level. This expandable list shows the proximity of where missing services can be found and the distance/travel time from the current community (Figure 11).

### Community Level Reports

**Figure 12:**
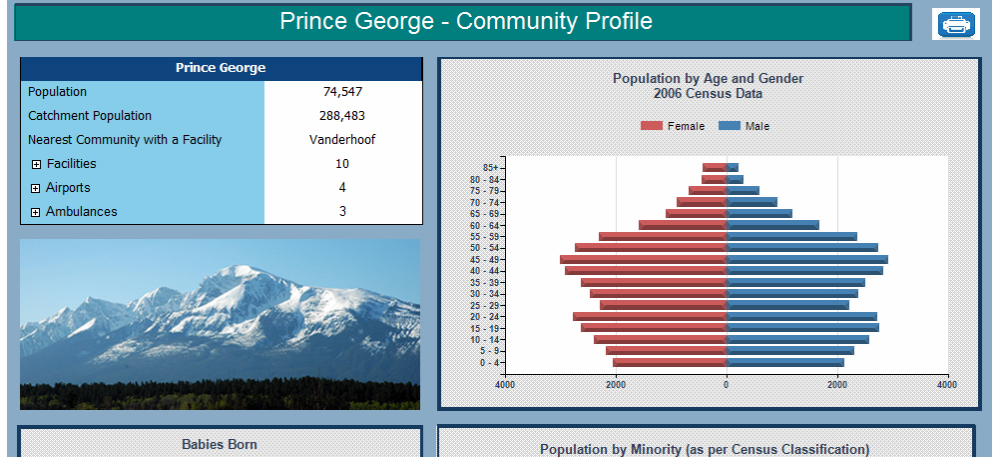
Community profile.

The Community Profile (Figure 12) summarizes demographic information, chronic conditions, hospitalization rates, available services, and other health-related metrics. It can be browsed from a Community report, which provides a list of communities, separated by their HSDA and grouped by LHA, thereby allowing direct access to the community profile reports.

**Figure 13:**
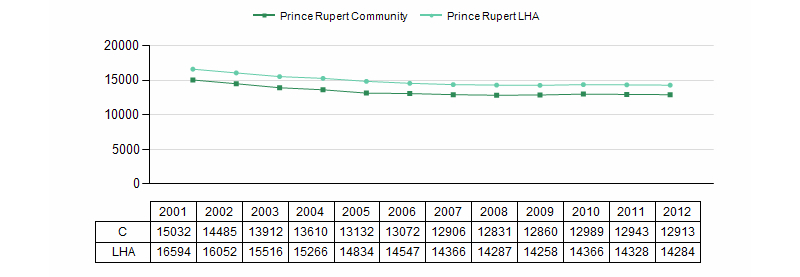
Population trend chart.

**Figure 14:**
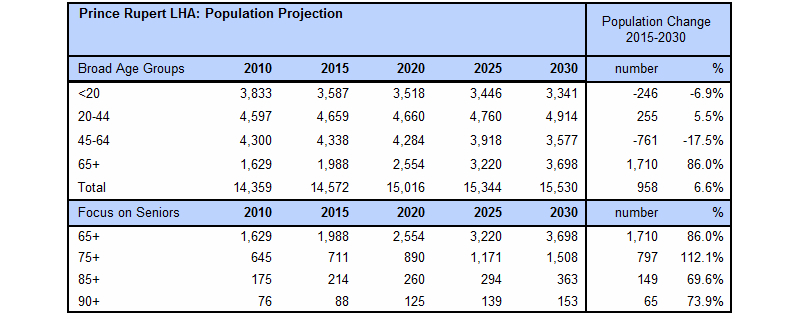
Population projection.

**Figure 15:**
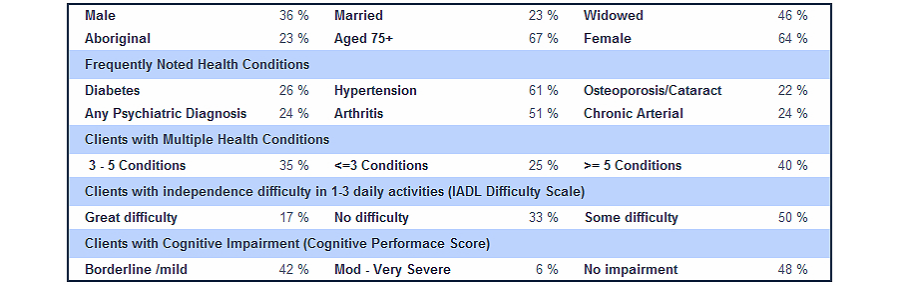
Senior residents profile.

For each community, there is a provision to access a comprehensive community health printable report, which contains a collection of tables, charts, and other relevant information including: Historical Population Information (Figure 13), Health Indicators, Population Forecasts (with a focus on seniors) (Figure 14), Births, Immunization Information, Vaccine Preventable Diseases, Chronic Diseases, Senior Resident Profile (Figure 15), and Facility Activity and Available Services.

### Other Reports

The reports illustrated in this paper are a small representative sample, due to space limitations. There are several other main and drill-down reports that provide various perspectives of the services availability. For instance, a community health report contains transfers/referrals information from/to the selected community in addition to charts and graphs that appear elsewhere in the application. These reports are printable and generally made available to communities. Similarly, many charts open a popup window instead of loading another report. These popups windows consist of descriptive charts or tables, definitions, and contain information about the source of data together with names of data analysts responsible for the information.

## Discussion

### Principal Findings

We have demonstrated how BI techniques and tools can be used in non-traditional areas of the health care environment to make informed decisions with reference to resource allocation and enhancement of the quality of patient care. The multidimensional cube allows analysis of data in several dimensions and reports are generated within seconds. The data can be kept up to date year round while preserving integrity during interim reporting. Originally, the data was updated annually due to the complexity of data collection and compilation. The versatility of reports is enhanced through parameterization, which allows values to be passed between sub-reports. The interaction of Web forms with the underlying database and cube allows for transparent data upload and integrity checks. The interactive reports provide users with valuable information such as proximity to location of available services, facilities with specific needs, comparative analysis, and tools for resource reallocation, if necessary. For privileged information, access controls have been implemented. The rural setting made this work more challenging because of the sparse geography and distance/travel times between facilities. Further, not all services are available in all communities, which requires identification of next best facility for repatriation of patients.

### Conclusions

The overall impact of the work presented in this paper spans a number of areas such as better allocation of available funds and better outcomes by making informed decisions regarding medical and personnel resource utilization. Though these benefits have not been quantified, it has already been observed that analysts’ time is now redirected to more effective surveillance activities and performance monitoring instead of collating data to manually generate reports.

It should also be noted that though the developed dashboard is not intended for real-time data, periodic surveillance reports can be generated on demand. Further, while the concept is applicable to all health authorities, it will be a challenge to have all jurisdictions collaborate and agree on a common architecture and/or report structures. Currently, the health planners and service providers internal to Northern Health are using the dashboard and planning is underway to have it accessible to the general public. The solution is modular and new datasets such as for smoking rates, teen pregnancies, HIV rates, immunization coverage, and vital statistical summaries can be easily integrated into the existing dashboard. The model can also be extended to other programs such as Home and Community Care, and Mental Health and Addictions. The next phase of this research is to determine how to incorporate services provided by non-NH providers such as Aboriginal Health Services.

## Abbreviations

BC: British Columbia
BI: business intelligence
CMG: case mix groups
ETL: extract-transform-load
GIS: geographic information system
HSDA: health service delivery area
KPI: key performance indicator
LHA: local health authority
NH: Northern Health
NHA: Northern Health Authority
OLAP: online analytical processing
SSIS: SQL Server Integration Services
